# The Involvement of Genes in Adolescent Depression: A Systematic Review

**DOI:** 10.3389/fnbeh.2015.00329

**Published:** 2015-12-21

**Authors:** Liangwei Xia, Shuqiao Yao

**Affiliations:** ^1^Medical Psychological Institute, Second Xiangya Hospital, Central South UniversityChangsha, Hunan, China; ^2^National Technology Institute of PsychiatryChangsha, Hunan, China

**Keywords:** adolescent, depression, gene, genetics, polymorphism, single nucleotide

## Abstract

Numerous studies have reported on the roles of genetic factors in the development of depression in adolescents and young adults. However, there are few systematic reviews that update our understanding of adolescent depression with the biological findings identifying the roles of gene expression and/or polymorphism(s). This review systematically summarized the findings that clearly identified the contribution of a gene to the risk of depression in adolescents between the ages of 10 and 19 years old and young adults between the ages of 20 and 25 years old. Data were obtained through searching PubMed, Embase, and Web of Science. A total of 47 studies on early adolescence and three studies on young adults were included in the current review. Most articles studied genes in the serotonergic system (*n* = 26), dopaminergic system (*n* = 3), and the Brain-derived neurotropic factor (BDNF) gene (*n* = 12). 92.3% of studies (24/26) identified positive associations of 5-HTTLPR polymorphism with depressive illness or depressive symptoms. 83.3% of studies (10/12) found positive association between *BDNF* Val66Met genotype and adolescent depressive symptoms. More studies should be conducted on the 18 genes reported in a few studies to clarify their roles in the risk for adolescent depression.

## Introduction

Depression is a common disorder affecting an estimated 350 million people worldwide. Long-lasting depression with moderate or severe intensity is a serious medical condition that can lead to suicide. An estimated 1 million deaths each year are related to suicide. Although treatments for depression are effective, fewer than half of all individuals with depression around the world (in some countries, fewer than 10%) take anti-depressants (WHO, 2014a). Barriers to effective care include a lack of resources, a lack of trained health care providers, and social stigma associated with mental disorders (WHO EMRO, 2014). Inaccurate assessment and incorrect diagnosis of depression in its early stages can also prevent the effective care of individuals with depression. It is commonly believed that depression is a result of the complex interaction of social, psychological, and biological factors. Numerous studies have reported the involvement of abnormal gene expression or single nucleotide polymorphisms (SNPs) of genes in the development of depression (Bufalino et al., 2013).

Depression is also the leading cause of disability in young people worldwide. An estimated nine percent of children and adolescents in the US are affected by depression (Dunn et al., 2011). Although the diagnostic criteria for children and adolescent depression are no different from those for adults, one epidemiological study showed that there are different risk factors for the onset of depression in young people and in adults (Jaffee et al., 2002). For example, depressive adolescents experienced more perinatal insults, caretaker instability, and criminality than adult-onset depressive patients. Also, depressive adolescents have more behavioral and socioemotional problems than the adult-onset patients. In contrast, the adult-onset patients experienced more sexual abuse in their childhood than depressive adolescents (Jaffee et al., 2002).

Moreover, studies in neuroscience have demonstrated that adolescence is a special period of development characterized by significant changes in the structure and connectivity of the brain, as well as changes in cognition and behavior (Cousins and Goodyer, 2015). These neurological changes may interplay between genes and the environment (Paus, 2013). Results from several family and twin studies suggest that etiologic genetics do exist in depression during adolescence (Rice, 2009). A few reviews or meta-analyses have also summarized the gene × environment interaction in adolescents with depression (Franić et al., 2010; Rice, 2010; Dunn et al., 2011). However, the age range for adolescence was not accurately defined. Heterogeneity within a group of depressive patients may be problematic for the development of theory, research, and treatment of depressive patients (Jaffee et al., 2002). It is therefore necessary to summarize the findings in adolescents with an accurately defined age range: (1) to update the findings on gene expression or polymorphism in adolescent depression and (2) to evaluate the value of all genes as a biomarker of adolescent depression. The WHO defines adolescence as having an age range of 10–19 years old and as a dynamic period with biological, social, and psychological changes (WHO, 2014b). In this study, the age range of 10–19 years was defined as adolescence while an age range of 20–25 years was defined as young adult for analysis.

The goal of the current review is to systematically analyze studies that tested the role of a gene in the development of depression or as a risk factor of depression in adolescents. We focused specifically on the findings and ultimately provide substantive conclusions on which gene expression or polymorphism could be a biomarker of depression in adolescents.

## Methods

### Eligibility Criteria

A systematic review of original studies on gene expression or genetic polymorphisms in adolescents with depression was conducted. Reports on gene expression or polymorphisms measured in peripheral blood or postmortem studies in adolescents were eligible for review. A study was included in the analysis when: (1) adolescents with depression or depressive symptoms fell into the range of 10–19 years old (WHO, 2014b) or young adult at an age of 20–25 years and (2) original research with the age range and gene being clearly identified. Studies were excluded if: (1) a study had subjects with age not in the two age ranges; (2) a study only provided a mean age; (3) A study contained anxious adolescents only; (4) studies that were conducted in animals or *in vitro*; or (5) review or studies that were not written in English.

### Information Sources

Studies were pulled from the electronic databases of PubMed, Embase, and Web of Science.

### Search Strategy

The primary search strategy was carried out using both the keywords and test words: “depression” and “adolescent” and “gene”; “depression” and “adolescent” and “polymorphism”; “depression” and “adolescent” and “genetics”; “depression” and “adolescent” and “genetic variants”; and “depression” and “adolescent” and “genotype”.

### Data Collection Process

**Figure 1:**
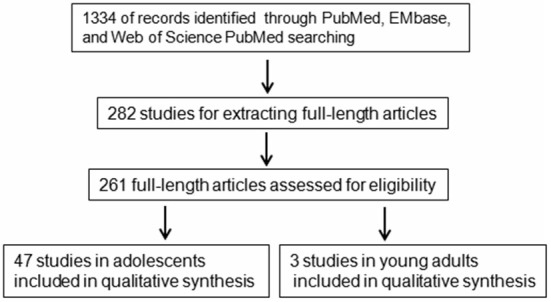
**A flowchart of literature search strategy.** A flowchart of the inclusions and exclusions of studies in the current study.

The abstract of each study was screened and the full-text articles of potentially relevant studies were then retrieved and assessed. Data were extracted from the retrieved papers by two authors (LX and SY) independently. Disagreements were resolved by discussion in a meeting that included several experts from within the Department. The study selection process was presented in a flow diagram (Figure 1).

### Data Items

Data on age range, study design, demographic characteristics of patients, and control individuals, gene expression or polymorphism, tissue type, and the results were extracted.

### Outcomes

The association of gene expression or polymorphisms with disease risk, severity of depressive symptoms, and treatment outcomes.

### Risk of Bias in Individual Studies

Risk of bias was assessed by evaluating choice of study design, selection of study population, allocation of control individuals, and quality of assay used.

### Data Synthesis

Data was summarized for each gene including the main outcomes or conclusion, positive and negative association with depression.

## Results

The literature search identified 1334 articles from PubMed, EMbase, and Web of Science using the keywords mentioned above after the removal of duplicates. The end date for the search is August 29, 2015. The abstracts of the 1334 articles were screened one by one and articles not written in English, review articles not for depression in adolescents, comments, and articles for animal or *in vitro* studies were excluded from this study. The studies where the gene name could not be identified in the abstract were also excluded from further analysis. A total of 282 studies were finally used for extracting full-length articles. However, only 261 full-length articles were downloaded for careful identification. Full-length articles of screening studies, studies in non-adolescents or with a mixed sample with the age beyond the range of 10–19 years for adolescents or 20–25 years for young adults, studies with only the mean age, studies without clearly identified gene expression or polymorphism, or others was excluded from the analysis. Finally, 47 full-length articles (Eley et al., 2004; Mamalakis et al., 2004; Burcescu et al., 2005; Miraglia del Giudice et al., 2006; Sjöberg et al., 2006; Geng et al., 2007; Hilt et al., 2007; Pandey et al., 2007, 2012; Feng et al., 2008; Aslund et al., 2009; Duncan et al., 2009; Goodyer et al., 2009, 2010; Guo and Tillman, 2009; Laucht et al., 2009; Nilsson et al., 2009; Nobile et al., 2009; Benjet et al., 2010; Brent et al., 2010; Mata et al., 2010; Nederhof et al., 2010; Uddin et al., 2010, 2011; Bouma et al., 2011; Hankin et al., 2011; Jenness et al., 2011; Mata and Gotlib, 2011; Thompson et al., 2011; Chen et al., 2012, 2013; Petersen et al., 2012; Buchmann et al., 2013; Bobadilla et al., 2013; Comasco et al., 2013; Cutuli et al., 2013; Kohen et al., 2013; Otten and Engels, 2013; Oppenheimer et al., 2013; Priess-Groben and Hyde, 2013; Stavrakakis et al., 2013; Banducci et al., 2014; Cicchetti and Rogosch, 2014; Cruz-Fuentes et al., 2014; Little et al., 2014; Zhang et al., 2015) in adolescents and three studies (Hammen et al., 2010; Starr et al., 2013; Thompson et al., 2014) in young adults were included in this study (Figure 1). The 47 studies in adolescence included 20 genes or gene families while the three studies in young adults included three genes (Table 1).

**Table 1:** **Summary of literature and outcomes in adolescents**.

Gene	Reference	Measurement	Association with depression
DRD2	Guo and Tillman (2009), Stavrakakis et al. (2013) and Zhang et al. (2015)	Polymorphism	1 no/2 yes
DRD4	Guo and Tillman (2009), Bobadilla et al. (2013) and Stavrakakis et al. (2013)	Polymorphism	1 no/2 yes
Catechol-O-methyltransferase (COMT)	Stavrakakis et al. (2013)	Polymorphism	no
Serotonin 2A	Stavrakakis et al. (2013)	Polymorphism	no
Monoamine oxidase A (MAOA)	Eley et al. (2004) and Stavrakakis et al. (2013)	Polymorphism	no
Tryptophan hydroxylase gene (TPH1/2)	Nobile et al. (2009), Stavrakakis et al. (2013) and Eley et al. (2004)	Polymorphism	1 no/2 yes
Serotonin transporter gene (5-HTT)	Eley et al. (2004), Sjöberg et al. (2006), Aslund et al. (2009), Goodyer et al. (2009), Goodyer et al. (2010), Laucht et al. (2009), Nobile et al. (2009), Benjet et al. (2010), Uddin et al. (2010), Uddin et al. (2011), Hankin et al. (2011), Jenness et al. (2011), Mata and Gotlib (2011), Petersen et al. (2012), Buchmann et al. (2013), Comasco et al. (2013), Cutuli et al. (2013), Kohen et al. (2013), Oppenheimer et al. (2013), Otten and Engels (2013), Priess-Groben and Hyde (2013), Stavrakakis et al. (2013), Banducci et al. (2014), Cicchetti and Rogosch (2014) and Little et al. (2014)	Polymorphism	24 yes/2 no
BDNF	Hilt et al. (2007), Duncan et al. (2009), Goodyer et al. (2010), Mata et al. (2010), Nederhof et al. (2010), Chen et al. (2012), Chen et al. (2013), Buchmann et al. (2013), Comasco et al. (2013), Stavrakakis et al. (2013), Cicchetti and Rogosch (2014) and Cruz-Fuentes et al. (2014).
FK506 binding protein 5 (FKBP5) gene	Brent et al. (2010)	Polymorphism	yes
Estrogen receptor α, β	Geng et al. (2007)	Polymorphism	no
Androgen receptor (AR)	Geng et al. (2007)	Polymorphism	no
Glucocorticoid receptor	Bouma et al. (2011)	Polymorphism	no
IL-1β/IL-6/TNF-α	Pandey et al. (2012)	Expression	yes
Neurotrophic tyrosine kinase receptor	Feng et al. (2008)	Linkage analysis	yes
CREB/CREB1	Burcescu et al. (2005) and Pandey et al. (2007)	Expression/Polymorphism	yes/no
Adipose polyunsaturated fatty acid gene	Mamalakis et al. (2004)	Expression	no
CART	Miraglia del Giudice et al. (2006)	Polymorphism	yes
OXTR	Thompson et al. (2011)	Polymorphism	yes
AP-2β	Nilsson et al. (2009)	Polymorphism	yes
HTR2A/2C	Eley et al. (2004)	Polymorphism	yes

Among the studies in early adolescence, only three articles reported the association between gene expression and depression in adolescents (Mamalakis et al., 2004; Pandey et al., 2007, 2012) and one study reported a linkage analysis (Feng et al., 2008). 91.5% (43/47) of articles reported the results of nucleotide polymorphism analysis of a gene in adolescents with depressive disease or depressive symptoms (Table 1). Most studies were related to neurotransmitter receptors and their associated metabolic enzymes, including the dopaminergic system (*DRD2, DRD4*, and *COMT*) and serotonergic system (serotonin 2A, *5-HTT, MAOA*, and *TPH*). *5-HTT* (*5-HTTLPR* polymorphism) is the most frequently examined gene (26 articles) followed by Brain-derived neurotropic factor (BNDF; 12 articles). Thirteen genes were only reported in one article. Seven genes (*COMT*, serotonin 2A, *MAOA*, estrogen receptor α and β, *AR*, glucocorticoid receptor, and adipose polyunsaturated fatty acid gene) showed no association with depression in adolescents. Four genes (*DRD2, DRD4, TPH1/2*, and *CREB/CREB1*) were reported by two or three articles with inconsistencies. The 26 studies on *5-HTTLPR* contained a total of 14,616 samples, and the 12 studies on *BDNF* contained a total of 7646 samples.

Approximately 92.3% (24/26) of studies for the association between *5-HTTLPR* polymorphism and depression in adolescents yielded a positive outcome (Table 2). Among the 26 studies for *5-HTTLPR* polymorphism, 22 studies analyzed the association between polymorphism and depressive symptoms (two studies showed negative findings), while four studies analyzed the role of *5-HTTLPR* polymorphism in depression. Only one study analyzed the association between *5-HTTLPR* polymorphism and the severity of depressive symptoms. It appears that *5-HTTLPR* polymorphism is associated with both the risk and severity of depression in adolescents. There was no study to compare the role of *5-HTTLPR* polymorphism in anxiety and depression. The association between *5-HTTLPR* polymorphism and different types of depression (i.e., anxiety vs. irritability vs. cognitive dysfunction) was not analyzed in this study because only a few studies analyzed the association between *5-HTTLPR* polymorphism and the cognitive dysfunction and the irritability.

**Table 2:** **Outcomes of studies on the relationship between 5-HTTLPR and depression in adolescents**.

Reference	Sample size	Main outcomes or conclusion
Stavrakakis et al. (2013)	1196	Adolescents’ depressive symptoms are not modified by 5-HTTLPR
Nobile et al. (2009)	607	Short alleles were associated with higher affective problems scores
Kohen et al. (2013)	192	The s/l vs. l/l genotype showed greater reduction in depression symptoms
Comasco et al. (2013)	1393	5-HTTLPR interacted with unfavorable environment in relation to depressive symptoms
Cutuli et al. (2013)	267	Positive G × E effects on depression were found
Priess-Groben and Hyde (2013)	309	Short allele confers susceptibility to stress for females with depression
Jenness et al. (2011)	200	5-HTTLPR predict depressive symptoms	
Otten and Engels (2013)	310	Cannabis use increases the risk of depression only in the presence of 5-HTTLPR short allele genotype
Uddin et al. (2011)	2574	The sl genotype carriers had* less* higher depressive symptom score
Goodyer et al. (2010)	401	5-HTTLPR short allele modify the risk of a new depressive episode associated with elevated morning salivary cortisol
Benjet et al. (2010)	78	Short alleles confers vulnerability to depressive symptoms in girls
Goodyer et al. (2009)	403	Episode of depression was increased in those with the “s” allele
Laucht et al. (2009)	309	LL genotype of 5-HTTLPR displayed significantly higher rates of depressive disorders and more depressive symptoms
Sjöberg et al. (2006)	200	Females carrying the short 5-HTTLPR allele tend to develop depressive symptoms
Nederhof et al. (2010)	1096	Interaction between 5-HTTLPR polymorphism and childhood adversities did not predict depression score
Cicchetti and Rogosch (2014)	1096	G × E interaction of 5-HTTLPR and maltreatment on depression symptoms
Little et al. (2014)	174	Structural abnormalities in the left hippocampus may be partly responsible for an indirect association between 5-HTTLPR genotype and depressive illness
Banducci et al. (2014)	222	Among girls, but not boys, each copy of the s allele of the 5-HTTLPR was related to increased depressive symptoms
Buchmann et al. (2013)	259	The carriers of the BDNF Met and 5-HTTLPR s allele are susceptible to depressive symptoms
Oppenheimer et al. (2013)	241	Youth with SS genotype of 5-HTTLPR experienced greatest increases in depressive symptoms when exposed to elevations in materal symptoms
Petersen et al. (2012)	436	Stress affect adolescents’ likelihood of experiencing depressed symptoms when they have a low serotonin TE (A/Gmodified5-HTTLPR) genotype
Mata et al. (2010)	50	Girls with homozygous for short 5-HTTLPR allele showed stronger association between depressive and bulimic symptoms the long allele
Hankin et al. (2011)	220	5-HTTLPR confers susceptibility to depression via stress reactivity
Uddin et al. (2010)	524	5-HTTLPR sl genotype is a risk of depressive symptom in adolescent male
Aslund et al. (2009)	1482	A GxE interaction effect of 5HTTLPR ss allele was found among girls, not boys
Eley et al. (2004)	377	A significant genotype-environmental risk interaction for 5HTTLPR in the risk of depression in girls only

*BDNF* was the second most frequently studied gene in adolescents. All 12 studies analyzed the association between *BDNF* Val66Met genotype/gene plasticity index and depressive symptoms. Among them, one study investigated the *BDNF* gene plasticity index in adolescents with depressive symptoms without observing any association. Eleven studies (Hilt et al., 2007; Duncan et al., 2009; Goodyer et al., 2010; Mata et al., 2010; Chen et al., 2012, 2013; Buchmann et al., 2013; Comasco et al., 2013; Stavrakakis et al., 2013; Cicchetti and Rogosch, 2014; Cruz-Fuentes et al., 2014) investigated the association between the *BDNF* Val66Met genotype and adolescent depressive symptoms with only one study showing no association (Nederhof et al., 2010; Table 3). However, no study analyzed the association between *BDNF* Val66Met genotype and severity of depressive symptoms. No study compared the role of *BDNF* Val66Met genotype in anxiety and depression. The association between *BDNF* Val66Met genotype and different types of depression was not analyzed in this study because no studies analyzed the *BDNF* Val66Met genotypes and irritability. Only one study investigated the association of *BDNF* Val66Met genotypes with cognitive dysfunction. Two studies reported the association of genetic variants in the dopaminergic system with adolescent depression (Bobadilla et al., 2013; Stavrakakis et al., 2013; Table 3).

**Table 3:** **Outcomes of studies on the relationship between BDNF and dopaminergic pathway and depression in adolescents**.

Reference	Sample size	Main outcomes or conclusion
Stavrakakis et al. (2013)	1196	Adolescents’ depressive symptoms are not modified by BDNF
Comasco et al. (2013)	1393	Depressive symptoms and depression were more common among carriers of either the Val/Val or Met genotypes
Goodyer et al. (2010)	401	*BDNF* (Val66Met) modify the risk of a new depressive episode associated with elevated morning salivary cortisol
Chen et al. (2012)	780	Interaction between *BDNF* Val66Met polymorphism and environmental stress on depression was observed
Nederhof et al. (2010)	1096	Depression score was not significantly predicted by interaction between BDNF Val66Met polymorphism and childhood adversities
Mata et al. (2010)	82	BDNF met allele moderate the relation between exercise and depressive symptoms
Duncan et al. (2009)	217	Val/Val genotype correlated with higher levels of depression symptoms
Hilt et al. (2007)	100	Val/Val genotype was associated with more depressive symptoms
Cicchetti and Rogosch (2014)	1096	G × G × E interaction of BDNF, *5-HTTLPR/CRHR1* and maltreatment on depression symptoms
Cruz-Fuentes et al. (2014)	246	Possession of BDNF Met allele was statistically linked with a resilient phenotype of major depression disorder
Buchmann et al. (2013)	259	The carriers of the BDNF Met and *5-HTTLPR* s allele are susceptible to depressive symptoms
Chen et al. (2013)	780	*BDNF* Val allele modulates the influence of environmental stress on depression
Stavrakakis et al. (2013)	1196	Adolescents’ depressive symptoms are not modified by COMT
Guo and Tillman (2009)	2286	DRD2*304/178 and DRD4*379/379 genotype are associated with a level of depressive symptoms
Bobadilla et al. (2013)	1882	DRD4 polymorphism is linked to comorbid marijuana use and depression

Only three studies investigated the gene polymorphism and the risk of depression in young adults at an age range of 20–25 years. Thompson et al. (2014) study found that OXTR (oxytocin receptor gene) polymorphism influences the development of depressive symptoms. Starr et al. (2013) study showed that *5-HTTLPR* S-allele can predict relative increases in probability of depression among boys. Hammen et al. (2010) study revealed that chronic family stress at age 15 predicted higher depression scores at age 20 among females with one or two S alleles of *5-HTT* gene (Table 4).

**Table 4:** **Summary of literature and outcomes in young adult**.

Gene	Reference	Measurement	Association with depression
OXTR	Thompson et al. (2014)	Polymorphism, *n* = 441	OXTR influences the development of depressive symptoms
5HTTLRP	Starr et al. (2013)	Polymorphism, *n* = 354	S-allele predicts relative increases in probability of depression among boys with low security
5HTT	Hammen et al. (2010)	Polymorphism, *n* = 346	Chronic family stress at age 15 predicted higher depression scores at 20 among those females with one or two S alleles

Risk of bias was evaluated for the study design, allocation of control individuals, and quality of assay methods. There was a wide variation in methods and analysis. Most studies did not include age or gender-matched control individuals. Most studies did not provide detailed information about current medical treatment in the full study sample. Most studies did not provide observer-based rating scores of depressive symptom severity.

## Discussion

Studies in twins, families, and populations have revealed the genetic influences on depression (Rice, 2009). Deregulated gene expression and specific functional genetic polymorphisms have been demonstrated to be risk factors of depression or to be associated with the severity of depressive symptoms (Dunn et al., 2011). Numerous studies have confirmed the existence of biological etiology in the development and progression of depression during childhood and adolescence (Dunn et al., 2011; Paus, 2013; Cousins and Goodyer, 2015). However, the published reviews did not summarize the up to date biological findings in only adolescents with defined age ranges. Heterogeneity within study subjects is a main concern for the research in depressive patients because the risk factors for the onset of depression in adolescents and in adults are different. Moreover, the depressive adolescents may experience more psychopathology, behavioral and socioemotional problems than adult-depressive patients (Jaffee et al., 2002). It is therefore important to summarize the biological findings in depressive patients with a defined age range. This study updated the genetic findings in adolescent depression with an age range of 10–19 years defined by WHO as adolescence and 20–25 years as young adult. This study demonstrated that genetic polymorphism or expression of 13 genes was associated, but seven tested genes were not associated with the risk or severity of depression in early adolescence, while the polymorphism of three genes was associated with the risk of depression in late adolesence. This review highlighted the role of several genes or gene families as risk factors for the development of depression in adolescents. This study improved our understanding of the etiology of adolescent depression, and also identified *5-HTTLPR* and *BDNF* Val66Met polymorphisms as the most studied biomarkers in adolescent depression.

Low serotonin-receptor levels in the brain have been widely recognized to be a key cause of depression. Polymorphisms of the 5-hydroxytryptamine (serotonin) transporter gene-linked polymorphic region (*5-HTTLPR*) have been widely demonstrated to be a risk factor for depression following adverse life experiences. In this review, a total of 26 articles reported an association between *5-HTTLPR* variations and depression in adolescents. About 92.3% of studies (*n* = 24) identified positive associations of 5-HTTLPR polymorphism with the risk for or susceptibility to depression, new depressive episodes, and more severe depressive symptoms. The most commonly examined polymorphism was the *5-HTTLPR* variable number tandem repeat (VNTR), which consists of the s/s, s/l, and l/l genotypes. In most studies, the short (S) allele or heterozygote genotype carriers (s/l) of 5-HTTLPR might experience a greater reduction in depressive symptoms over time compared with adolescents with the *5-HTTLPR* l/l genotype (Table 2). A recent meta-analysis of the association between the *5-HTTLPR*, stress, and the development of depression contained 81 studies with a mean age from 9–77 years. A significant relationship between the short form of the *5-HTTLPR* and depression was confirmed (*p* = 0.0000009). However, nearly 26% of the 81 studies failed to show any significant association, and four studies even showed opposite results (Sharpley et al., 2014). Only two studies investigated the *5-HTTLPR* polymorphism and the risk of depression in young adults at an age range of 20–25 years. These two studies showed a positive association between *5-HTTLPR* S-allele and the increased probability of depression in adolescents (Hammen et al., 2010; Starr et al., 2013). These findings suggest that *5-HTTLPR* polymorphism is a risk factor for both early onset and late onset depression.

Tryptophan hydroxylase-2 (*TPH2*) gene has been acknowledged for many years as the only form of tryptophan hydroxylase (TPH) responsible for the synthesis of serotonin in the brain and peripheral tissues. A recent report suggested that *TPH2* gene expression in the dorsal raphe nuclei of depressed suicidal patients is upregulated [14]. This review included 2 studies on *TPH2* in adolescents (Nobile et al., 2009; Stavrakakis et al., 2013). However, adolescents’ depressive symptoms are not associated with the gene plasticity index of *TPH2* (Stavrakakis et al., 2013). Monoamine oxidase A is a monoamine oxidase encoded by the *MAOA* gene that preferentially deaminates norepinephrine, epinephrine, serotonin, and dopamine. Studies in adolescents demonstrated that depressive symptoms are not associated with gene plasticity index of the *MAOA* gene (Stavrakakis et al., 2013), whereas gene × environment interaction of the *5-HTTLPR* was further moderated by *MAOA* activity level (Nobile et al., 2009).

BDNF is the most abundant neurotrophin in the mammalian central nervous system, and reduced BDNF level in the hippocampus has been revealed to be related to the onset of depression (Bai et al., 2012). A single nucleotide polymorphism (SNP) in the *BDNF* gene (Val66Met) has been shown to influence the activity of the BDNF protein and cause subsequent memory impairment and harm avoidance (Jiang et al., 2005). A significant interaction between *BDNF* Val66Met and life stress in depression was widely observed in adults (Hosang et al., 2014). In this review, the *BDNF* gene is the second most frequently studied gene in adolescents with depression. Two studies on the *BDNF* gene plasticity index and BDNF level showed controversial outcomes. The depressive symptoms were not associated with gene plasticity index of the *BDNF* gene in adolescents (Stavrakakis et al., 2013), whereas *BDNF* mRNA level correlated with symptom improvement in adult patients with depression (Cattaneo et al., 2010). Seven studies investigated the association between *BDNF* Val66Met polymorphism and adolescent depression. Two studies showed no association between *BDNF* Val66Met polymorphism and depression score, but 10 studies found significant correlations between BDNF polymorphism and adolescent depression. A recent meta-analysis of the interaction between the *BDNF* Val66Met polymorphism and stress in depression contained 22 studies with a mean age range from 8.85–65 years. Results showed that the Met allele of *BDNF* Val66Met significantly moderates the relationship between life stress and depression (*p* = 0.03; Hosang et al., 2014). This review only contained three studies with an age range from 9–19 years. Moreover, a meta analysis of genes and suicide in 16 studies showed that hypermethylation of *BDNF* is associated with individuals that died of suicide (Lockwood et al., 2015). The involvement of genetic polymorphisms in the *BDNF* gene in peripheral tissues of patients with depression may reflect changes in the BDNF level in their brain. The Val66Met polymorphism has been demonstrated to impair the packaging and secretion of BDNF and subsequently reduces hippocampal volume and impairs memory (Kimpton, 2012; Harrisberger et al., 2015).

Dopamine is a major neurotransmitter in the central nervous system, and dysregulation of the dopaminergic-system is widely reported in people with depression. The hypofunction of the mesolimbic dopaminergic pathway has been linked to anhedonia, one of the major symptoms of depression (Leggio et al., 2013). Studies that directly link genetic polymorphisms in the dopaminergic pathway to depression are abundant but show inconsistent findings (Lawford et al., 2006). For example, Lawford et al study demonstrated that patients with DRD2*A1/A2 genotype had significantly higher depression scores compared to those with the DRD2*A2/A2 genotype (Lawford et al., 2006). An interaction between *DAT1* polymorphism and perceived maternal rejection can influence the onset of depressive disorder and suicidal ideation (Haeffel et al., 2008). A significant association between the 48 bp repeat polymorphism of *DRD4* and depression was reported (Manki et al., 1996). In contrast, Frisch et al. (1999) found no association of polymorphisms in *DRD4, DAT1*, and *COMT* with depression. Kirov et al. (1999) found that six dopaminergic genes (*DBH, DAT1, COMT, DRD2, DRD3*, and *DRD5*) played no role in bipolar disorder. However, the roles of genetic polymorphisms in the dopaminergic pathway in adolescent depression have not been summarized. In this study, three studies reported associations between genetic variants in the dopaminergic system and adolescent depression (Bobadilla et al., 2013; Stavrakakis et al., 2013). Significant associations between *DRD2, DRD4*, and *COMT* polymorphisms and the risk of depression, as well as the severity of depressive symptoms, were reported.

Selective serotonin reuptake inhibitors (SSRIs) are the first line treatment for depression in the clinic. These inhibitors work by increasing serotonin levels in the brain to counteract the low serotonin-receptor levels. A meta-analysis has demonstrated that the *5-HTTLPR* long-allele carriers had higher probability of response than patients with short-allele homozygotes of *5-HTTLPR* with heterogeneity effect (Serretti et al., 2007). No study investigated the effect of *5-HTTLPR* polymorphism on the efficacy of SSRIs treatment at an age range from 9–19 years. Rotberg et al. (2013) study in 83 children and adolescents aged 7–18 years showed that the *5-HTTLPR* ss genotype was associated with a poorer clinical response to citalopram with regards to depressive symptoms. A recent meta analysis showed that the BDNF Met carriers had a better response rate to SSRI than Val/Val, while Met/Val carriers had a weak effect of response to SSRIs than Val/Val carriers (Yan et al., 2014). Based on the report from Hammen et al. (2010) study that patients younger than 18 years old have an increased risk of suicidal thoughts or behaviors (4% with SSRIs vs. 2% with placebo) with an antidepressant (Gordon and Melvin, 2013), FDA suggests that adolescents taking SSRIs must be closely monitored to reduce the risk for suicide. However, the risk is small and the risk hasn’t been replicated very well in studies of adolescent depression treated with SSRIs. For example, Hetrick et al. (2012) study showed that no significant increase in suicide-related outcomes was observed in children and adolescents after using individual SSRIs, such as paroxetine, fluoxetine, sertraline, citalopram, and escitalopram.

In conclusion, there are more published positive associations between the genetic polymorphisms in the serotoninergic system, dopaminergic system, and the Val66Met polymorphism of BDNF with adolescent depression. However, some biases should be considered: (1) the publication bias for positive findings; (2) numerous negative findings may be unpublished; (3) a bias for investigations of these three polymorphisms first because of the high profile papers that brought them to attention; and (4) the polymorphisms can be easily genotyped. Although the expression or polymorphism of 18 genes has been reported to be or not be associated with the risk of depression, they were reported by only a few studies. Therefore, further studies are needed to accurately identify their roles in depression in adolescents.

## Author Contributions

LX: data extraction, data analysis, and manuscript writing. SY: study design, data extraction, data analysis, critical revision of manuscript, and a guarantor of the review.

## Funding

This study is supported by grants from the National Natural Science Foundation of China (81471384 to SY).

## Conflict of Interest Statement

The authors declare that the research was conducted in the absence of any commercial or financial relationships that could be construed as a potential conflict of interest.
